# Psychopathic personality traits in 5 year old twins: the importance of genetic and shared environmental influences

**DOI:** 10.1007/s00787-016-0899-1

**Published:** 2016-09-28

**Authors:** Catherine Tuvblad, Kostas A. Fanti, Henrik Andershed, Olivier F. Colins, Henrik Larsson

**Affiliations:** 1 School of Psychology, Law and Social Work, Örebro University, Örebro, Sweden; 2 Department of Psychology, University of Southern California, Los Angeles, CA USA; 3 Department of Psychology, University of Cyprus, Nicosia, Cyprus; 4 Departments of Child and Adolescent Psychiatry, Curium-Leiden University Medical Center, Leiden, The Netherlands; 5 Department of Medical Epidemiology and Biostatistics, Karolinska Institute, Solna, Sweden; 6 Karolinska Institute Center for Neurodevelopmental Disorders, Solna, Sweden; 7 School of Medical Sciences, Örebro University, Örebro, Sweden

**Keywords:** Psychopathic personality traits, Heritability, Teacher ratings, Childhood

## Abstract

There is limited research on the genetic and environmental bases of psychopathic personality traits in children. In this study, psychopathic personality traits were assessed in a total of 1189 5-year-old boys and girls drawn from the Preschool Twin Study in Sweden. Psychopathic personality traits were assessed with the Child Problematic Traits Inventory, a teacher-report measure of psychopathic personality traits in children ranging from 3 to 12 years old. Univariate results showed that genetic influences accounted for 57, 25, and 74 % of the variance in the grandiose–deceitful, callous–unemotional, and impulsive–need for stimulation dimensions, while the shared environment accounted for 17, 48 and 9 % (n.s.) in grandiose–deceitful and callous–unemotional, impulsive–need for stimulation dimensions, respectively. No sex differences were found in the genetic and environmental variance components. The non-shared environment accounted for the remaining 26, 27 and 17 % of the variance, respectively. The three dimensions of psychopathic personality were moderately correlated (0.54–0.66) and these correlations were primarily mediated by genetic and shared environmental factors. In contrast to research conducted with adolescent and adult twins, we found that both genetic and shared environmental factors influenced psychopathic personality traits in early childhood. These findings indicate that etiological models of psychopathic personality traits would benefit by taking developmental stages and processes into consideration.

## Introduction

Psychopathy is a multifaceted syndrome often described as a constellation of affective (e.g., lack of remorse or guilt, shallow affect, callous/lack of empathy), interpersonal (e.g., glib/superficial charm, grandiose self-worth, manipulative), behavioral and antisocial (e.g., poor behavior control, impulsive, inability to accept responsibility for one’s actions) features [17, 38]. Psychopathy is related to a range of negative and dysfunctional outcomes including substance use, criminal behavior, psychopathology (e.g., borderline personality disorder) [21, 40, 42, 44, 56], and social maladjustment such as lower educational performance, unemployment and poor social relationships [4, 19, 44, 71]. The prevalence of psychopathy has been estimated to be between 0.6 and 4 % in the general population, with a higher proportion of males to females [65, 75]. Despite the low prevalence, these individuals are believed to account for a large portion of all serious crimes and their recidivism rate is higher than for other offenders [8, 38, 56]. Furthermore, psychopathy is considered to be a neurodevelopmental disorder rooted early in life [22, 33, 35, 36, 41, 52]. Identifying individuals with psychopathic personality traits early in development is, therefore, crucial for intervention efforts, especially since these traits have been linked to early engagement in criminal activities (e.g., [39, 58, 62]). To date, there has been very limited research on the genetic and environmental etiology of psychopathic personality traits in young children. This study aimed to bridge this research gap by investigating the extent of genetic influences on these traits in boys and girls and to investigate whether shared environmental influences also play a significant role.

Recent twin studies report that heritable factors have a moderate to high influence, non-shared environmental factors have a small to moderate influence, and shared environmental factors have little or no importance explaining the variance in psychopathic personality (for reviews: [66, 72]). This has been found among adolescents (e.g., [5, 9, 25, 30, 47, 48, 64, 69, 70]), as well as among adults (e.g., [5, 10, 11, 44]). However, a more mixed pattern has been found across the few studies that have included children (i.e., participants 12 years of age or younger; see Table 1 for a summary). Please also note that the majority of the studies summarized in Table 1 have only examined the affective (or callous–unemotional) traits. For example, an early study by Viding et al. [76] using DeFries–Fulker extreme analysis showed that the heritability of callous–unemotional traits was 67 % in a sample of 7-year-old twins. Furthermore, Bezdjian et al. used the Child Psychopathy Scale [51] to assess psychopathic personality traits in a set of 9–10 year old twins. The affective–interpersonal factor was primarily influenced by genetic factors with slight sex differences (boys 64 %; girls 49 %), with the non-shared environment contributing 36 % in boys and 44 % in girls. Similarly, the impulsive–antisocial factor was primarily influenced by genetic factors (boys 46 %; girls 58 %), with the non-shared environment contributing 53 % in boys and 37 % in girls. There were little and non-significant influence from the shared environment [6]. Ficks et al. included children as young as 4 years in their study (age range 4.4–17.8 years, age-corrected analysis) and the antisocial process screening device [31] was used to assess psychopathic personality traits. For callous–unemotional traits, genetic influences explained 49 % of the variance, shared environment 19 % and the non-shared environment 32 %; for narcissism, genetic influences explained 63 % and the non-shared environment 37 %; and for impulsivity, additive and non-additive genetic influences contributed 61 % in boys and 74 % in girls, with the non-shared environment contributing the remaining variance.

**Table 1:** Genetic (A), shared environmental (C) (or dominant, D), and non-shared environmental (E) estimates for psychopathic personality traits in children (i.e., participants 12 years of age or younger)—a summary from previous twin studies

References	Sample (*N*)	Psychopathy measure	Informant	Age (years)	Psychopathy dimension	A	C/(D)	E
Bezdjian et al. [6]	RFAB^a^ (1219 twins)	Child Psychopathy Scale (CPS) [50]	Caregiver rated (>90 % biological Mothers)	9–10	Affective–interpersonal factor (boys)	0.64 (0.49 to 0.72)	0 (0.00 to 0.11)	0.36 (0.28–0.47)
					Affective–interpersonal factor (girls)	0.49 (0.21 to 0.65)	0.06 (0.00 to 0.30)	0.44 (0.35–0.56)
					Impulsive–antisocial factor (boys)	0.46 (0.22 to 0.58)	0.01 (0.00 to 0.195)	0.53 (0.41–0.66)
					Impulsive–antisocial factor (girls)	0.58 (0.25 to 0.70)	0.04 (0.00 to 0.34)	0.37 (0.29–0.48)
Frick and Hare [31]	Georgia Twin Study^c^ (885 twin pairs)	Antisocial process screening device [31]	Mother rated	4.4–17.8	Callous–unemotional	0.49^d^	0.19	0.32
					Narcissism	0.63		0.37
					Impulsivity (boys)	0.21	0.40	0.39
					Impulsivity (girls)	0.26	0.48	0.26
Viding et al. [76]	TEDS^b^ (612 + 234 + 210 twins)	Three antisocial process screening device [31] and four Strengths and Difficulties Questionnaire [37] items were used to assess callous–unemotional traits	Teacher rated	7	Elevated levels of callous–unemotional traits	0.67^e^ (0.47 to 0.87)	0.06 (−0.23 to 0.35)	
					Elevated levels of antisocial behavior and callous–unemotional traits	0.81 (0.50 to 1.12)	0.05 (0.00 to 0.72)	
					Elevated levels of antisocial behavior w/o callous–unemotional traits	0.30 (−0.10 to 0.70)	0.34 (−0.40 to 1.08)	
Larsson et al. [49]	TEDS^b^ (352 + 234 twins)	All items from callous–unemotional dimension of the antisocial process screening device [31] and two items from the Strengths and Difficulties Questionnaire [37] were used to assess callous–unemotional traits	Teacher rated	7	Callous–unemotional traits and elevated levels of antisocial behavior	0.68^e^ (0.42 to 0.95)	0.00 (−0.82 to 0.18)	
					Callous–unemotional traits w/o elevated levels of antisocial behavior	0.80 (0.51 to 1.03)	0.00 (−77 to 0.23)	
Viding et al. [80]	TEDS^b^ (140 probands in 88 twin pairs, and 174 probands in 144 twin pairs)	All items from callous–unemotional dimension of the antisocial process screening device [31] and two items from the Strengths and Difficulties Questionnaire [37] were used to assess callous–unemotional traits	Teacher rated	9	Antisocial behavior and callous–unemotional traits	0.75^e^ (0.45 to 1.06)	0.00 (−1.63 to 1.27)	
					Antisocial behavior only	0.53 (0.13 to 0.92)	0.00 (−0.83 to 0.64)	
					Antisocial behavior, hyperactivity and callous–unemotional traits	0.71 (0.24 to 1.18)	0.00 (−1.66 to 1.36)	
					Antisocial behavior and hyperactivity	0.36 (−0.14 to 0.86)	0.00 (−0.76 to 0.71)	
Humayun et al. [43]	TEDS^b^ (627 + 119 twins)	Three antisocial process screening device [31] and four Strengths and Difficulties Questionnaire [37] items were used to assess callous–unemotional traits	Teacher rated	7	Elevated levels of callous–unemotional	0.75^e^ (0.58 to 0.92)	−0.02 (−1.22 to 2.38)	
					Elevated levels of callous–unemotional and Anxiety	0.66 (0.33 to 0.95)	0.05 (−1.62 to 2.28)	
Fontaine et al. [29]	TEDS^c^ (9462)	Three Antisocial Process Screening Device [31] and four Strengths and Difficulties Questionnaire [37] items were used to assess callous–unemotional traits	Teacher rated	7, 9, 12	Stable high (boys) (girls)	0.78 (0.42 to 0.88)	0.01 (0.00 to 0.35)	0.21 (0.12–0.34)
						0.00 (0.00 to 0.57)	0.75 (0.35 to 0.90)	0.25 (0.07–0.48)
					Increasing (boys) (girls)	0.58 (0.12 to 0.72)	0.03 (0.00 to 0.41)	0.39 (0.28–0.53)
						0.26 (0.00 to 0.70)	0.47 (0.08 to 0.74)	0.27 (0.16–0.43)
					Decreasing (boys) (girls)	0.61 (0.35 to 0.72)	0.02 (0.00 to 0.23)	0.37 (0.28–0.49)
						0.54 (0.23 to 0.85)	0.26 (0.00 to 0.53)	0.20 (0.13–0.29)
					Stable low (boys + girls)	0.68 (0.52 to 0.81)	0.08 (0.00 to 0.21)	0.24 (0.19–0.30)

^a^The University of Southern California Risk Factors for Antisocial Behavior (RFAB) twin study. Other papers using the same sample and measure but not necessarily examining the influence of genetic and environmental factors on psychopathic personality traits are not included in Table 1. For example, a study examining the genetic and environmental overlap between psychopathic traits and aggression at age 9–10 [7], a study examining the relationship between psychopathic traits and autonomic responses during the countdown task at age 9–10 [74], a study examining the relationship between psychopathic traits and anticipatory fear skin conductance responses at age 9–10 [73], a study examining the relationship between psychopathic traits and skin conductance orienting response at age 9–10 [45]

^b^Twins Early Development Study. Other papers using the same sample and measure but not necessarily examining the influence of genetic and environmental factors on psychopathic personality traits are not included in Table 1. For example, a study examining the genetic and environmental overlap between callous–unemotional traits and conduct problems at age 7 [79], a study examining the genetic and environmental overlap between callous–unemotional traits at age 7 and autistic traits at age 8 [57], a study examining the relationship among negative parental discipline, conduct problems and callous–unemotional traits at age 7 and 12 [78], a study examining the relationship among dimensions of psychopathy (callous–unemotional traits, narcissism, impulsivity) and cognitive abilities at age 9 [26], a study using growth mixture modeling to identify four trajectories of callous–unemotional traits (stable high, increasing, decreasing, and stable low) at ages 7, 9, 12 [28], a study examining the relationship between peer victimization and trajectories of callous–unemotional traits (i.e., stable high, increasing, decreasing and stable low) at ages 7, 9, 12 [27]. See also reviews: a review on callous–unemotional traits, including twin studies examining its genetic and environmental etiology [81], a review on callous–unemotional traits and antisocial behavior [77]

^c^Georgia Twin Study. Other papers using the same sample and measure and are not included in Table 1 include for example a study examining the factor structure of the antisocial process screening device [20]

^d^95 % confidence intervals for the ACE estimates nor standard errors were not reported

^e^Estimates of group heritability and group shared environment were calculated using the DeFries–Fulker extreme analysis regression model [18]

In sum, studies examining the genetic and environmental etiology of psychopathic personality traits in young children are scarce. The few studies that do exist have produced diverse findings showing a moderate to strong heritability; the role of the shared environment on these traits is mixed, and whether or not the genetic and environmental estimates vary across sex is unclear. Using data from a population-based sample of Swedish 5-year-old twins this study had the following goals: (1) to examine the genetic and environmental etiology of the three psychopathic personality dimensions—grandiose–deceitful, a callous–unemotional, and impulsive–need for stimulation-assessed with the Child Problematic Traits Inventory (CPTI; [13]), which was designed to be used specifically among young children; (2) to examine whether the genetic and environmental etiology of the three psychopathic personality dimensions is comparable in boys and girls; and (3) to examine how much of the phenotypic correlation among these dimensions that are accounted for by genetic and environmental influences.

## Method

### Participants and procedure

This study used data from the Preschool Twin Study in Sweden (PETSS) project. The overall aim of PETSS was to examine how genetic and environmental factors in early childhood contribute to cognition, emotional regulation and behavioral problems. Parents of all twins born in Sweden between January, 2004 and May, 2005 were identified through the Swedish population-based medical birth register and contacted 1 month prior to their twins’ 5th birthday. Thus, all children in PETSS were 5 years old at study start. Questionnaires were mailed to parents and pre-school teachers of 1261 twin pairs (*n* = 2522 children). Non-responders were approached with up to three reminders. Parents were approached separately, resulting in 828 (65 %) responses from the mothers, and 698 (55 %) responses from the fathers. Mother and/or father ratings were available from 879 twin pairs (*n* = 1758 children). The teacher-rated questionnaires had a response rate of 54 % 686 twin pairs (*n* = 1372 children). The PETSS project was evaluated and approved by an ethics committee at the Karolinska Institute (#2007-1034). For more information on study protocol and procedures: http://ki.se/meb/petss, and [12].

The main focus of this study was to examine genetic and environmental influences on psychopathic personality traits assessed with the CPTI [13]. The CPTI was completed only by teachers and data were available from a total of *n* = 1189 children (591 boys; 598 girls), Table 2.

Typically in Sweden, children start pre-school (day care) around or soon after their first birthday. They remain in pre-school for a period of approximately 4 years. They go to Kindergarten in the fall of the year that they turn 6 years. The PETSS questionnaire was mailed out and completed by participating pre-school teachers close to the twins’ fifth birthday. Of the participating teachers, 3 % reported that they had known the twins less than 6 months, 12 % reported that they had known the twins between 6 and 12 months, 11 % reported that they had known the twins between 13 and 18 months, 12 % reported that they had known the twins between 19 and 24 months, and 62 % reported that they had known the twins more than 24 months.

**Table 2:** Descriptive statistics and twin correlations for the psychopathic personality dimensions grandiose–deceitful, callous–unemotional and impulsive–need for stimulation at age 5, teacher ratings

	Boys	Girls	
Grandiose–deceitful (mean, SD)	1.41, 0.52 *n* = 591	1.33, 0.49 *n* = 598	*t*_(1187)_ 3.04, *p* = 0.0025
Callous–unemotional (mean, SD)	1.60, 0.65 *n* = 591	1.38, 0.51 *n* = 598	*t*_(1187)_ 6.61, *p* < 0.001
Impulsive–need for stimulation (mean, SD)	2.11, 0.72 *n* = 591	1.91, 0.66 *n* = 598	*t*_(1187)_ 5.17, *p* < 0.001

	MZ	DZ	MZ	DZ	OSDZ
Grandiose–deceitful	0.79*	0.25*	0.75*	0.44*	0.50*
Callous–unemotional	0.72*	0.66*	0.66*	0.58*	0.54*
Impulsive–need for stimulation	0.82*	0.25*	0.80*	0.34*	0.54*

*MZ* monozygotic, *DZ* dizygotic, *OSDZ* opposite sex

* *p* < 0.05, transformed data

### Measures

Psychopathic personality traits were assessed with the Child Problematic Traits Inventory (CPTI) [13, 14, 16, 63]. The CPTI has the following response format: 1 = ‘Does not apply at all’; 2 = ‘Does not apply well’; 3 = ‘Applies fairly well’; and 4 = ‘Applies very well’. Respondents were instructed to rate each child on how he/she usually and typically behaves rather than based on how he or she behaves at the moment. CPTI contains 28 items which have been found to load on three factors, and this three factor structure has been identified in PETSS across boys and girls [15], as well as in another Swedish sample across boys and girls and across age [13]. The grandiose–deceitful factor score includes eight items (e.g., lies often to avoid problems; seems to see himself/herself as superior compared to others), the callous–unemotional factor score includes 10 items (e.g., seldom expresses sympathy for others; usually does not seem to share others’ joy and sorrow), and the impulsive–need for stimulation factor score includes ten-items (e.g., likes change and that things happen all the time; often has difficulties with awaiting his/her turn). The CPTI three factor scores showed excellent internal consistency (all Cronbach’s alphas in the present study >0.89). In terms of external validity, the three factors exhibited positive and significant correlations with teacher and parent rated variables of interest in PETSS, including conduct problems, attention–deficit/hyperactivity (ADHD) symptoms, aggression, and fearlessness [15]. All three scores were log-transformed to approximate a normal distribution.

### Statistical analyses

#### Descriptive statistics and correlations

Descriptive statistics, including means and standard deviations, were first computed for three psychopathic personality dimensions: grandiose–deceitful, callous–unemotional, and impulsive–need for stimulation, as well as their phenotypic correlations.

#### Twin modeling

In the twin design, data from monozygotic (MZ) and dizygotic (DZ) twins are used to decompose the variance in a measured trait to genetic and environmental components. MZ twins share their common environment and they are assumed to share 100 % of their genes. DZ twins also share their common environment and they are assumed to share about 50 % of their genes. By comparing the resemblance between MZ and DZ twins, the variance of a measured trait can be divided into additive genetic factors (A), shared environmental factors (C), and non-shared environmental factors (E). Shared environmental factors refer to non-genetic influences that contribute to similarity within pairs of twins, whereas non-shared environmental factors refer to experiences that make siblings dissimilar [55].

To get a first indication of the underlying sources of variance in grandiose–deceitful, callous–unemotional, and impulsive–need for stimulation dimensions, comparisons were made among twin correlations (Twin-1–Twin-2 correlations). A DZ correlation approximately half the value of the MZ correlation would indicate the presence of additive genetic effects, whereas a DZ correlation more than half an MZ correlation indicates the presence of both genetic and shared environmental effects [55].

#### Univariate modeling

Univariate models were fit to grandiose–deceitful, callous–unemotional, and impulsive–need for stimulation separately to estimate the relative contributions of genetic factors (A), shared environmental factors (C), and non-shared environmental factors (E, plus error). To test for sex differences in the variance components, a model in which the genetic and environmental effects were allowed to differ between boys and girls were compared against a model in which the estimates were constrained to be equal. A saturated model, which estimates the variances, covariances, and means were first fit and used as a baseline model to which all subsequent models were compared.

#### Bivariate modeling

A bivariate Cholesky decomposition was fit to estimate how much of the phenotypic correlation that is due to genetic and environmental influences between grandiose–deceitful, callous–unemotional and impulsive–need for stimulation, also referred to as bivariate heritability, bivariate shared environment and bivariate non-shared environment. These estimates are proportions and range from 0 to 1. They provide information regarding the extent to which the phenotypic correlation between two traits is mediated by genetic and/or environmental factors.

All genetic models were fit with the structural equation program Mx [54]. The goodness of fit was compared through the difference in the Chi-square statistic (*χ* ^2^), where a significant *χ* ^2^ indicates that the model with less number of parameters fits the data worse. Akaike Information Criterion (AIC) [1] and Bayesian Information Criterion (BIC) [60] were also used to determine fit, where increasingly negative values correspond to increasingly better fitting models.

## Results

### Descriptive statistics and correlations

There were significant mean differences between boys and girls, with boys having higher mean values for grandiose–deceitful, callous–unemotional, and impulsive–need for stimulation. The pattern of the twin correlations indicates that genetic and shared environmental influences are important for the three psychopathic personality dimensions, Table 2.

### Univariate genetic analysis

Univariate model-fitting results for grandiose–deceitful, callous–unemotional, and impulsive–need for stimulation are displayed in Table 3. A low DZ twin correlation (in boys for grandiose–deceitful; boys and girls for impulsive–need for stimulation, Table 2) may be due to non-additive genetic effects, such as epistasis or dominance [54]. A model estimating additive genetic (A) effects, non-additive genetic (D) effects and non-shared environmental (E) effects was, therefore, first tested. However, the full ACE model (Model 2 in Table 3) was found to fit better than the ADE model (grandiose–deceitful: AIC 768.632, BIC −2210.855; impulsive–need for stimulation: AIC 694.695, BIC −2247.824).

**Table 3:** Univariate genetic results and parameter estimates for the psychopathic personality dimensions grandiose–deceitful, callous–unemotional and impulsive–need for stimulation at age 5, teacher ratings

Model #	Overall fit							Parameter estimates		
	−2log	*df*	AIC	BIC	*χ* ^2^-diff	Δ*df*	*p*	A	C	E
Grandiose–deceitful										
1. Saturated model	3109.314	1172	765.314	−2194.920						
2. ACE boys ≠ girls	3116.395	1181	754.395	−2220.173	7.081	9	0.629			
3. ACE boys = girls	3120.357	1184	752.357	−2227.789	11.043	12	0.525	0.57 (0.39–0.75)	0.17 (0.001–0.32)	0.26 (0.21–0.33)
Callous–unemotional										
1. Saturated model	3018.635	1172	674.635	−2240.259						
2. ACE boys ≠ girls	3023.728	1181	661.728	−2266.506	5.093	9	0.826			
3. ACE boys = girls	3028.018	1184	660.018	−2273.959	9.383	12	0.670	0.25 (0.10–0.40)	0.48 (0.35–0.60)	0.27 (0.22–0.33)
Impulsive–need for stimulation										
1. Saturated model	3035.396	1172	691.396	−2231.879						
2. ACE boys ≠ girls	3049.638	1181	687.638	−2253.552	14.242	9	0.114			
3. ACE boys = girls	3050.872	1184	682.872	−2262.532	15.476	12	0.216	0.74 (0.59–0.86)	0.09 (0.00–0.23)	0.17 (0.14–0.21)

*AIC* Akaike’s Information Criterion, *BIC* Bayesian Information Criterion, *χ* ^*2*^ *-diff* difference in log-likelihoods between models, *df* degrees of freedom, *A* additive genetic, *C* shared environment, *E* non-shared environment

The full ACE model described the data better than the baseline saturated model (Model 2, grandiose–deceitful: *χ* ^2^ = 7.081, *df* = 9, *p* = 0.629, callous–unemotional: *χ* ^2^ = 5.093, *df* = 9, *p* = 0.826, impulsive–need for stimulation: *χ* ^2^ = 14.242, *df* = 9, *p* = 0.114); Model 2 also had smaller AIC and BIC. A model constraining genetic and environmental components to be equal in boys and girls provided a better fit than the full ACE model (Model 3, grandiose–deceitful: *χ* ^2^ = 3.962, *df* = 3, *p* = 0.266, callous–unemotional: *χ* ^2^ = 4.29, *df* = 3, *p* = 0.232, impulsive–need for stimulation: *χ* ^2^ = 1.234, *df* = 3, *p* = 0.745). Genetic influences accounted for 57, 25, and 74 % of the phenotypic variance for grandiose–deceitful, callous–unemotional and impulsive–need for stimulation, respectively; shared environmental factors accounted for 17, 48, and 9 % (n.s.), and non-shared environmental factors (including error) accounted for the remaining variance, 26, 27, and 17 %, respectively.

### Bivariate genetic analysis

A bivariate Cholesky decomposition was next fit to data. The Cholesky decomposition fit the data better than a saturated model (*χ* ^2^ = 76.143, *df* = 69, *p* = 0.260). Similar to the univariate analyses, the genetic and environmental variance components could be constrained to be equal in boys and girls (*χ* ^2^ = 21.157, *df* = 18, *p* = 0.272). The phenotypic correlations were moderate to high across the three psychopathic personality dimensions (Table 3). The phenotypic correlations between grandiose–deceitful, callous–unemotional, and impulsive–need for stimulation were primarily accounted for by genetic and shared environmental influences (Table 4).

**Table 4:** Proportion of the phenotypic correlations between grandiose–deceitful, callous–unemotional, and impulsive–need for stimulation accounted for by genetic (A), shared environmental (C) and non-shared environmental (E) factors

	*Phenotypic correlation*	bivh^2^ (95 % CI)	bivc^2^ (95 % CI)	bive^2^ (95 % CI)
Grandiose–deceitful/callous–unemotional	0.66	0.25 (0.05–0.43)	0.52 (0.36–0.67)	0.23 (0.17–0.32)
Grandiose–deceitful/impulsive–need for stimulation	0.61	0.56 (0.40–0.72)	0.26 (0.12–0.40)	0.18 (0.12–0.24)
Callous–unemotional/impulsive–need for stimulation	0.54	0.39 (0.20–0.59)	0.44 (0.26–0.60)	0.17 (0.11–0.24)

*bivh* ^*2*^ bivariate heritability, *bivc* ^*2*^ bivariate shared environment, *bive* ^*2*^ bivariate non-shared environment

## Discussion

This study aimed to investigate the genetic and environmental sources among three psychopathic personality dimensions, grandiose–deceitful, callous–unemotional and impulsive–need for stimulation in a community sample of 5-year-old children assessed by teachers. There are three main points of interest for discussion in this study. First, familial influences (i.e., genetic and/or shared environment) explained the majority of variance in grandiose–deceitful, callous–unemotional and impulsive–need for stimulation. Second, no sex differences were found in the genetic and environmental variance components. Third, the proportions of the phenotypic correlations among these dimensions were mainly mediated by genetic and shared environmental influences.

Similar to Ficks et al. [25], our univariate analyses indicated that genetic and shared environmental influences primarily explained the variance in the callous–unemotional dimension, and that a large genetic influence was important for impulsive–need for stimulation. We also found that genetic and shared environmental influences explained the variances in grandiose–deceitful, whereas Ficks et al. found that mainly genetic influences were important for narcissism. This discrepancy in findings between our study and Ficks et al. could partly be explained by methodological differences in that we were using the CPTI rated by teachers and they were using the antisocial process screening device rated by mothers. As genetic influences on psychopathic personality traits may vary across the ways in which these traits are measured, in terms of both informant and instrument used [69], more research examining the genetic and environmental etiology of these traits in early childhood is warranted. Also, Ficks et al. [25] age-corrected their data (age range 4.4–17.8 years), whereas we used a sample of 5-year-old twins.

Further, callous–unemotional traits have previously received attention [25, 32, 41], and recently a callous–unemotional-based specifier for the diagnosis of conduct disorder has been added in the fifth edition of the diagnostic and statistical manual of mental disorders [2]. Our finding of a moderate genetic (25 %) influence and higher shared environmental (48 %) influence on the callous–unemotional dimension is in sharp contrast to findings by Viding et al. [76] who found a high heritability (67 %) for antisocial behavior in the presence of callous–unemotional traits as reported by teachers in a sample of 7 year old twins, and no influence from the shared environment. Thus, we found that both genetic and shared environmental factors contributed to callous–unemotional traits at age five. Shared environmental risk factors may include family related factors (e.g., neglect, prenatal stressors) or contextual factors in the surrounding community [53, 68]. Our finding agree with prior work linking environmental factors to callous–unemotional traits and studies suggesting that interventions focusing on environmental stimuli may be effective in reducing callous–unemotional traits (for a review: [34]). The moderate genetic influence in our sample for callous–unemotional traits might also be related to heterogeneity within these traits, with subgroups showing differences in behavioral and physiological measures of anxiety and fear reactivity (e.g., [23, 24]).

The three dimensions grandiose–deceitful, callous–unemotional and impulsive–need for stimulation were all moderately correlated. The proportions of these phenotypic correlations were mainly accounted for by genetic and shared environmental influences. Again, these findings provide support for the importance of both genetic and shared environmental influences in psychopathic personality traits in young children.

The significant shared environmental influences in particular for callous–unemotional traits identified in our sample of 5-year-old twins are of great importance. Typically, a pattern of decreasing shared environment and a concomitant increase in heritability over the course of development is found; this has been reported for several phenotypes including personality traits, cognitive abilities, and aggression [59]. It will be interesting to follow the twins included in this study across development to see if a similar pattern will emerge for the shared environment on psychopathic personality traits. Then again, the bulk of literature on psychopathy has shown little or no influence of the shared environment (e.g., [66, 72]); however, the majority of previous research has been conducted on adolescent or adult twin samples. Of note, twin studies typically have low power to detect shared environmental influences relative to genetic influences. Shared environmental influences can also be confounded with for example the effects of assortative mating or passive gene-environment correlation (*rGE*) [46]. Thus, part of the shared environment we found could be explained by the fact that the same teacher was rating both twins in a pair [3]. In our case, 97 % of the participating twin pairs went to the same pre-school class, and 85 % were rated by the same teacher. Studies of children typically rely on parent or teacher reports; it is, therefore, possible that the shared environment found in these studies and in our study is partly an artifact of rater bias. This suggests that future studies are needed examining how genetic and environmental factors influence psychopathic personality traits in children, and it will be interesting to see if they can replicate our finding of a shared environmental component.

We also found higher mean values for psychopathic personality traits in boys than girls across all three psychopathic personality dimensions, indicating that these traits are somewhat more prevalent among boys than girls. Higher mean values for psychopathic personality traits have also been found among males than females across incarcerated and community samples [75]. However, no differences in the magnitude in genetic and environmental variance components were found across boys and girls and the variance components could be constrained to be equal. This finding is in contrast to Bezdjian et al. [7], who found significant sex differences across 9–10 year old boys and girls, with the affective–interpersonal factor showing higher heritability in boys and the impulsive–antisocial factor showing higher heritability in girls. Similarly, Ficks et al. [25] found sex differences in the Impulsivity dimension, with a higher heritability in girls. Our findings suggest that despite sex differences on a mean level, the underlying genetic and environmental etiology of these traits appears to be similar for both boys and girls. This would in turn indicate that there are specific circumstances (biological) or experiences (social, environmental) that may lead to greater expression of psychopathic personality traits in boys. Future research needs to determine which specific factors that contributes to the sex difference in prevalence.

### Limitations

A few limitations in this study must be considered when interpreting these findings. First, we examined the genetic and environmental influences on psychopathic personality traits in a community sample of young twins. Our results may not be generalizable to children in clinical settings. We only had one time point, future research need to investigate how genetic and environmental factors influence change in these traits from early childhood through adolescence and whether the shared environment that we found will decrease across development. There are several assumptions related to the classical twin design [59], for example, the heritability estimate is time and population specific. A more detailed discussion of these and other assumption in the twin design in relation to psychopathology can be found elsewhere [67].

## Conclusions

In contrast to research conducted with adolescent and adult twins, we found that both genetic and shared environmental influences are of importance for psychopathy personality traits in childhood. The phenotypic correlations between three dimensions of psychopathic personality grandiose–deceitful, callous–unemotional, and impulsive–need for stimulation were primarily accounted for by common genetic and common shared environmental influences. This highlights the importance of considering all three dimensions of psychopathic personality simultaneously in clinical work as well as in future research, see also [13, 61]. These findings further indicate that etiological models of psychopathic personality should take developmental stages and processes into consideration. This evidence is important for prevention efforts, suggesting that preventions designed to reduce the development of psychopathic personality traits can be successful if administered during the preschool developmental period.

## References

[1] Akaike AC (1987). Factor analysis and AIC. Psychometrika.

[2] American Psychiatric Association (2013). Diagnostic and statistical manual of mental disorders.

[3] Baker LA, Jacobson KC, Raine A, Lozano DI, Bezdjian S (2007). Genetic and environmental bases of childhood antisocial behavior: a multi-informant twin study. J Abnorm Psychol.

[4] Beaver KM, da Silva Costa C, Poersch AP, Freddi MC, Stelmach MC, Connolly EJ, Schwartz JA (2014). Psychopathic personality traits and their influence on parenting quality: results from a nationally representative sample of Americans. Psychiatr Q.

[5] Beaver, K. M., Vaughn, M. G., and Delisi, M. (2013). Nonshared environmental effects on adulthood psychopathic personality traits: results from a monozygotic twin difference scores analysis. Psychiatr Q **(Epub ahead of print)** 10.1007/s11126-013-9255-5 23378034

[6] Bezdjian S, Raine A, Baker LA, Lynam D (2011). Psychopathic personality in children: genetic and environmental contribution. Psychol Med.

[7] Bezdjian S, Tuvblad C, Raine A, Baker LA (2011). The genetic and environmental covariation among psychopathic personality traits, and reactive and proactive aggression in childhood. Child Dev.

[8] Blair JR, Mitchell DA, Blair K (2007). The psychopath, emotion and the brain.

[9] Blonigen DM, Hicks BM, Krueger RF, Patrick CJ, Iacono WG (2005). Psychopathic personality traits: heritability and genetic overlap with internalizing and externalizing psychopathology. Psychol Med.

[10] Blonigen DM, Hicks BM, Krueger RF, Patrick CJ, Iacono WG (2006). Continuity and change in psychopathic traits as measured via normal-range personality: a longitudinal-biometric study. J Abnorm Psychol.

[11] Brook M, Panizzon MS, Kosson DS, Sullivan EA, Lyons MJ, Franz CE, Kremen WS (2010). Psychopathic personality traits in middle-aged male twins: a behavior genetic investigation. J Personal Disord.

[12] Burt SA, Larsson H, Lichtenstein P, Klump KL (2012). Additional evidence against shared environmental contributions to attention–deficit/hyperactivity problems. Behav Genet.

[13] Colins OF, Andershed H, Frogner L, Lopez-Romero L, Veen V, Andershed AK (2014). A new measure to assess psychopathic personality in children: the Child Problematic Traits Inventory. J Psychopathol Behav Assess.

[14] Colins OF, Fanti K, Larsson H, Anckarsäter H (2016) Psychopathic traits in early childhood: further validation of the Child Problematic Traits Inventory. Assessment:1–16 **(epub ahead of print)** 10.1177/1073191115624544 26733308

[15] Colins OF, Kostas AF, Larsson H, Andershed H (2015) Psychopathic traits in early childhood: further validation of the Child Problematic Traits Inventory **(in preparation)** 10.1177/1073191115624544 26733308

[16] Colins OF, Veen V, Venstra M, Frogner L, Andershed H (2016). The Child Problematic Traits Inventory in a Dutch General Population sample of 3- to 7-year-old children. Eur J Psychol Assess.

[17] Cooke DJ, Michie C (2001). Refining the construct of psychopathy: towards a hierarchical model. Psychol Assess.

[18] DeFries JC, Fulker DF (1985). Multiple regression analysis of twin data. Behav Genet.

[19] DeLisi M, Vaughn M, Beaver KM, Wexler J, Barth AE, Fletcher JM (2011). Fledgling psychopathy in the classroom: ADHD subtypes, psychopathy, and reading comprehension in a community sample of adolescents. Youth Violence Juv Justice.

[20] Dong L, Wu H, Waldman ID (2014). Measurement and structural invariance of the antisocial process screening device. Psychol Assess.

[21] Douglas KS, Vincent GM, Edens JF, Patrick CJ (2006). Risk for criminal recidivism: the role of psychopathy. Handbook of psychopathy.

[22] Fairchild G, van Goozen SH, Calder AJ, Goodyer IM (2013). Research review: evaluating and reformulating the developmental taxonomic theory of antisocial behaviour. J Child Psychol Psychiatry.

[23] Fanti KA, Demetriou CA, Kimonis ER (2013). Variants of callous–unemotional conduct problems in a community sample of adolescents. J Youth Adolesc.

[24] Fanti KA, Panayiotou G, Lazarou C, Michael R, Georgiou G (2016). The better of two evils? Evidence that children exhibiting continuous conduct problems high or low on callous–unemotional traits score on opposite directions on physiological and behavioral measures of fear. Dev Psychopathol.

[25] Ficks CA, Dong L, Waldman ID (2014). Sex differences in the etiology of psychopathic traits in youth. J Abnorm Psychol.

[26] Fontaine N, Barker ED, Salekin RT, Viding E (2008). Dimensions of psychopathy and their relationships to cognitive functioning in children. J Clin Child Adolesc Psychol.

[27] Fontaine NM, Hanscombe KB, Berg MT, McCrory E, Viding E (2016). Trajectories of callous–unemotional traits in childhood predict different forms of peer victimization in adolescence. J Clin Child Adolesc Psychol.

[28] Fontaine NM, McCrory EJ, Boivin M, Moffitt TE, Viding E (2011). Predictors and outcomes of joint trajectories of callous–unemotional traits and conduct problems in childhood. J Abnorm Psychol.

[29] Fontaine NM, Rijsdijk FV, McCrory EJ, Viding E (2010). Etiology of different developmental trajectories of callous–unemotional traits. J Am Acad Child Adolesc Psychiatry.

[30] Forsman M, Lichtenstein P, Andershed H, Larsson H (2008). Genetic effects explain the stability of psychopathic personality from mid- to late adolescence. J Abnorm Psychol.

[31] Frick PJ, Hare RD (2001). *Antisocial Process Screening Device* Toronto.

[32] Frick PJ, Moffitt TE (2010). A proposal to the DSM-V childhood disorders and the ADHD and disruptive behavior disorders work groups to include a specifier to the diagnosis of conduct disorder based on the presence of callous–unemotional traits.

[33] Frick PJ, Ray JV (2015). Evaluating callous–unemotional traits as a personality construct. J Personal.

[34] Frick PJ, Ray JV, Thornton LC, Kahn RE (2014). Can callous–unemotional traits enhance the understanding, diagnosis, and treatment of serious conduct problems in children and adolescents? A comprehensive review. Psychol Bull.

[35] Gao Y, Glenn AL, Schug RA, Yang Y, Raine A (2009). The neurobiology of psychopathy: a neurodevelopmental perspective. Can J Psychiatry.

[36] Gao Y, Raine A (2010). Successful and unsuccessful psychopaths: a neurobiological model. Behav Sci Law.

[37] Goodman R (1997). The strengths and difficulties questionnaire: a research note. J Child Psychol Psychiatry.

[38] Hare RD (2003). The Hare psychopathy checklist-revised (PCL-R).

[39] Hemphill JF, Templeman R, Wong S, Hare RD, Cooke DJ, Forth AE, Hare RD (1998). Psychopathy and crime: recidivism and criminal careers. Psychopathy: Theory, research and implications for society.

[40] Hemphälä M, Hodgins S (2014). Do psychopathic traits assessed in mid-adolescence predict mental health, psychosocial, and antisocial, including criminal outcomes, over the subsequent 5 years?. Can J Psychiatry.

[41] Herpers PC, Rommelse NN, Bons DM, Buitelaar JK, Scheepers FE (2012). callous–unemotional traits as a cross-disorders construct. Soc Psychiatry Psychiatr Epidemiol.

[42] Hicks BM, Vaidyanathan U, Patrick CJ (2010). Validating female psychopathy subtypes: differences in personality, antisocial and violent behavior, substance abuse, trauma, and mental health. Personal Disord.

[43] Humayun S, Kahn RE, Frick PJ, Viding E (2014). Callous–unemotional traits and anxiety in a community sample of 7-year-olds. J Clin Child Adolesc Psychol.

[44] Hunt E, Bornovalova MA, Patrick CJ (2014). Genetic and environmental overlap between borderline personality disorder traits and psychopathy: evidence for promotive effects of factor 2 and protective effects of factor 1. Psychol Med.

[45] Isen J, Raine A, Baker LA, Dawson ME, Bezdjian S, Lozano DI (2010). Sex-specific association between psychopathic traits and electrodermal reactivity in children. J Abnorm Psychol.

[46] Keller MC, Medland SE, Duncan LE (2010). Are extended twin family designs worth the trouble? A comparison of the bias, precision, and accuracy of parameters estimated in four twin family models. Behav Genet.

[47] Larsson H, Andershed H, Lichtenstein P (2006). A genetic factor explains most of the variation in the psychopathic personality. J Abnorm Psychol.

[48] Larsson H, Tuvblad C, Rijsdijk FV, Andershed H, Grann M, Lichtenstein P (2007). A common genetic factor explains the association between psychopathic personality and antisocial behavior. Psychol Med.

[49] Larsson H, Viding E, Plomin R (2008). Callous–unemotional traits and antisocial behavior. Crim Justice Behav.

[50] Lynam DR (1997). Pursuing the psychopath: capturing the fledgling psychopath in a nomological net. J Abnorm Psychol.

[51] Lynam DR (2002). Fledgling psychopathy: a view from personality theory. Law Hum Behav.

[52] Miller JD, Lynam DR (2015). Psychopathy and personality: advances and debates. J Personal.

[53] Murray J, Farrington DP (2010). Risk factors for conduct disorder and delinquency: key findings from longitudinal studies. Can J Psychiatry.

[54] Neale MC, Boker SM, Xie G, Maes H (2003). Mx: statistical modeling.

[55] Neale MC, Cardon LR (1992). Methodology for genetic studies of twins and families.

[56] Neumann CS, Hare RD (2008). Psychopathic traits in a large community sample: links to violence, alcohol use, and intelligence. J Consult Clin Psychol.

[57] O’Nions E, Tick B, Rijsdijk F, Happé F, Plomin R, Ronald A, Viding E (2015). Examining the genetic and environmental associations between autistic social and communication deficits and psychopathic callous–unemotional traits. PLoS One.

[58] Pechorro P, Maroco J, Gonçalves RA, Nunes C, Jesus SN (2014). Psychopathic traits and age of crime onset in male juvenile delinquents. Eur J Criminol.

[59] Plomin R, DeFries JC, McClearn GE, McGuffin P (2008). Behavioral genetics.

[60] Raftery AE (1995). Bayesian model selection in social research. Sociol Methodol.

[61] Salekin RT (2016). Psychopathy in childhood: toward better informing the DSM–5 and ICD-11 conduct disorder specifiers. Personal Disord Theory Res Treat.

[62] Smith SS, Newman JP (1990). Alcohol and drug abuse/dependence disorders in psychopathic and nonpsychopathic criminal offenders. J Abnorm Psychol.

[63] Somma A, Andershed H, Borroni S, Fossati A (2016). The validity of the child problematic trait inventory in 6–12 year old Italian children: further support and issues of consistency across different sources of information and different samples. J Psychopathol Behav Assess.

[64] Taylor A, Loney BR, Bobadilla L, Iacono WG, McGue M (2003). Genetic and environmental influences on psychopathy trait dimensions in a community sample of male twins. J Abnorm Child Psychol.

[65] Thompson DF, Ramos C, Willett JK (2014). Psychopathy: clinical features, developmental basis and therapeutic challenges. J Clin Pharm Ther.

[66] Tuvblad C, Vaughn MG, DeLisi M (2014). Genetic influences on antisocial behavior over the life course. Handbook of biosocial criminology.

[67] Tuvblad C, Baker LA (2011). Human aggression across the lifespan: genetic propensities and environmental moderators. Adv Genet.

[68] Tuvblad C, Bezdjian S, Raine A, Baker LA (2013). Psychopathic personality traits and negative parent-to-child affect: a longitudinal cross-lag twin study. J Crim Justice.

[69] Tuvblad C, Bezdjian S, Raine A, Baker LA (2014). The heritability of psychopathic personality in 14 to 15 years old twins, multi-rater and multi-measure approach. Psychol Assess.

[70] Tuvblad C, Wang P, Bezdjian S, Raine A, Baker LA (2015). Psychopathic personality development from ages 9 to 18: genes and environment. Dev Psychopathol.

[71] Ullrich S, Farrington DP, Coid JW (2008). Psychopathic personality traits and life-success. Personal Individ Differ.

[72] Waldman ID, Rhee S, Patrick CJ (2006). Genetic and environmental influences on psychopathy and antisocial behavior. Handbook of psychopathy.

[73] Wang P, Baker LA, Gao Y, Raine A, Lozano DI (2012). Psychopathic traits and physiological responses to aversive stimuli in children aged 9–11 years. J Abnorm Child Psychol.

[74] Wang P, Gao Y, Isen J, Tuvblad C, Raine A, Baker LA (2015). Genetic covariance between psychopathic traits and anticipatory skin conductance responses to threat: evidence for a potential endophenotype. Dev Psychopathol.

[75] Verona E, Vitale J, Patrick CJ (2006). Psychopathy in women. Handbook of psychopathy.

[76] Viding E, Blair JR, Moffitt TE, Plomin R (2005). Evidence of substantial genetic risk for psychopathy in 7-year-olds. J Child Psychol Psychiatry.

[77] Viding E, Fontaine NM, McCrory EJ (2012). Antisocial behaviour in children with and without callous–unemotional traits. J R Soc Med.

[78] Viding E, Fontaine NM, Oliver BR, Plomin R (2009). Negative parental discipline, conduct problems and callous–unemotional traits: monozygotic twin differences study. Br J Psychiatry.

[79] Viding E, Frick PJ, Plomin R (2007). Aetiology of the relationship between callous–unemotional traits and conduct problems in childhood. Br J Psychiatry.

[80] Viding E, Jones AP, Frick PJ, Moffitt TE, Plomin R (2008). Heritability of antisocial behaviour at 9: do callous–unemotional traits matter?. Dev Sci.

[81] Viding E, McCrory EJ (2012). Genetic and neurocognitive contributions to the development of psychopathy. Dev Psychopathol.

